# Supplementary material for: Circadian Analysis of the Mouse Cerebellum Proteome
Source: Int J Mol Sci. 2019 Apr 15;20(8):1852. doi: 10.3390/ijms20081852 (PMC6515515; doi:10.3390/ijms20081852)
Supplement: Supplementary file 1 [file ijms-20-01852-s001.zip › Suppl Fig1_Plumel et al.docx]

**Supplementary Figure S1.** **Representative 2D gel image of proteins in the cerebellum of mice**

Shown are the protein spots exhibiting significant cosinor *p* values (*p*<0.05) for the Mesor, Amplitude, and Acrophase (groups ZT5, ZT11 ZT17, ZT23), except Spot N°42 that exhibited a *p* value for Amplitude of only 0.06.
